# Exosomal miRNA-16-5p Derived From M1 Macrophages Enhances T Cell-Dependent Immune Response by Regulating PD-L1 in Gastric Cancer

**DOI:** 10.3389/fcell.2020.572689

**Published:** 2020-11-30

**Authors:** Zhengtian Li, Bing Suo, Gang Long, Yue Gao, Jia Song, Mengzhe Zhang, Baiyu Feng, Ce Shang, Dawei Wang

**Affiliations:** ^1^Department of General Surgery, The First Affiliated Hospital of Harbin Medical University, Harbin, China; ^2^General Hospital of Heilongjiang Province Land Reclamation Bureau, Harbin, China

**Keywords:** gastric cancer, M1 macrophage, microRNA-16-5p, T cell immune response, PD-L1

## Abstract

Macrophages have an affinity to developing tumors and have been shown to play a role in tumor combat and immune surveillance. However, the exact mechanism by which macrophages participate in the anti-tumor immune response remains unclear. Hence, the current study aimed to identify the effect of macrophages on gastric cancer (GC) cells via exosomes. Paired cancerous, tumor-adjacent, and non-cancerous stomach tissues were initially from 68 GC patients. T cells were isolated from peripheral blood mononuclear cells (PBMCs) obtained from both the GC patients as well as the healthy donors. Next, the exosomes were isolated from LPS and IFN-γ-induced PBMCs (M1 macrophages) and co-cultured with human GC cells. Another co-culture system comprised of CD3^+^ T cells and exosomes-treated GC cells was then performed. BALB/c mice and NOD/SCID nude mice were prepared for effects of exosomal miR-16-5p on tumor growth and anti-tumor immune response in GC *in vivo*. A relationship between M1 macrophages and the poor survival of GC patients was identified, while they secreted exosomes to inhibit GC development and activate a T cell-dependent immune response. Our results revealed that miR-16-5p was transferred intercellularly from M1 macrophages to GC cells via exosomes and targeted PD-L1. M1 macrophage-derived exosomes containing miR-16-5p were found to trigger a T cell immune response which inhibited tumor formation both *in vitro* and *in vivo* by decreasing the expression of PD-L1. Taken together, the key findings of the current study suggest that M1 macrophage-derived exosomes carrying miR-16-5p exert an inhibitory effect on GC progression through activation of T cell immune response via PD-L1. Our study highlights the promise of M1 macrophages as a potential cell-based therapy for GC treatment by increasing miR-16-5p in exosomes.

## Introduction

Gastric cancer (GC) is a malignancy characterized by the growth of neoplastic tumor cells in the stomach As at 2015, GC was ranked as the second most commonly diagnosed cancer as well as the second leading cause of cancer related death in China (Chen et al., 2016). *Helicobacter pylori* represents one of the chief causative factors of GC, accounting for approximately 65∼80% of new GC cases on an annual basis (Kusters et al., 2006; de Martel et al., 2012). Other known risk factors include age, cigarette smoking, obesity, and dietary factors (Karimi et al., 2014). Tumor resection at the early stage of GC is often accompanied with high rates of survival while poor patient outcomes and survival are often the result of advanced stage GC, often due to metastatic GC cell migration to distant tissues and lymph nodes (Thrumurthy et al., 2015). Although there are various standard treatments approaches including surgery, endoscopic mucosal resection and chemoradiation, all of which are widely applied, the emergence of novel therapies such as targeted therapy and immunotherapy have been highlighted in literature (Digklia and Wagner, 2016). Tumor cells can evade the immune system, which is mediated by combination of tumor associated antigens (TAA) and immune checkpoints (Yousefi et al., 2017). Immunotherapy employs the use of antibodies that are capable of specifically blocking immune checkpoints which help to enhance T cell surveillance of tumor cells. More recently, immunotherapy approaches targeting PD1, PDL1, and CTLA4 have all been successfully applied in GC, with largely promising outcomes (Bonotto et al., 2017).

PD1 and PDL1 are immune checkpoints both of which are located on the cellular membrane and are capable of regulating the T cell receptor (TCR). PDL1 is expressed by a wide variety of cells including that of cancer cells and has been shown to inhibit cellular antigen presentation. PD1 preferentially appears in immune cells such as NK, B, and T cells (Bonotto et al., 2017). The interaction between PDL1 and PD1 has been widely reported to interfere with the TCR signaling transduction of T cells. Existing literature has revealed that monocytes such as macrophages can secrete immune factors that are able to regulate B, T, and NK cells. Recent studies have suggested macrophage-derived exosome carrying non-coding RNAs and immune factors can also control the immune effectors either by immune inhibition or immune activation (Veerman et al., 2019). For instance, exosomes generated by activated macrophages carry polarized M1 or M2 mRNAs and miRNAs (Garzetti et al., 2014). Furthermore, a correlation between miR-16 and cancer progression such as breast cancer and lung cancer has been reported (Sromek et al., 2017; Usmani et al., 2017). In GC, miR-16 has been identified as an antioncogenic factor that acts to inhibit the proliferation and migration of GC cells by targeting SALL4 (Jiang and Wang, 2018). Furthermore, miR-16 targets PDL1 and breaks the interaction between PDL1 and PD1 in prostate cancer, thus improving the radiotherapy via T cell activation (Jiang and Wang, 2018). The regulation of miR-16 on PD1/PDL1 axis in GC is still unknown. Hence, the current study aimed to elucidate the regulation of M1-derived exosomes carrying miR-16-5p on T cell.

## Materials and Methods

### Ethics Statement

Written informed consent was obtained from all patients prior to enrollment into the study. Study protocols were approved by the Ethics Committee of The First Affiliated Hospital of Harbin Medical University, with the protocol performed in strict accordance with the ethical principles for medical research involving human subjects of the Helsinki Declaration. All animal experiments were performed in strict accordance with the recommendations of the Guide for the Care and Use of Laboratory Animals of the National Institutes of Health. The animal experimental protocol was approved by the Institutional Animal Care and Use Committee of The First Affiliated Hospital of Harbin Medical University. Animal experiments were performed based on idea of minimizing animal number and minimizing the pains experienced by the animals.

### Study Subjects

A total of 68 GC patients (46 male and 22 female, aged 31–79 years with a mean age of 55 years) who had undergone excision procedures at The First Affiliated Hospital of Harbin Medical University between January 2013 and December 2015 were enrolled into the study. Based on tumor, lymph node metastasis and tumor node metastasis (TNM) grading criteria of the 7th edition of International Union Against Cancer, there were 20 cases at stage I, 15 cases at stage II, and 33 cases at stage III. GC, tumor-adjacent, and non-cancerous stomach tissues (5 cm away from the tumor) and peripheral blood were all collected from patients with GC. All enrolled patients were yet to have received any chemotherapy or radiotherapy prior to the operation. Patients diagnosed with infectious disease, autoimmune disease, or multiple primary cancers were excluded. A follow-up visit was performed in order to determine the overall survival (OS) rate of GC patients by means of phone call or follow-up review until December 2018. Furthermore, the peripheral blood from 40 healthy volunteers (16 male and 24 female, aged 32–78 years with a mean age of 57.35 years) was used as the peripheral blood control.

### Isolation of Macrophages and T cells

As previously described (Wang et al., 2017), GC and non-tumor tissues were made into single cell suspension and stained by anti-human antibodies against CD68, CD11c, and CD206. Fluorescence activated cell sorter FACSAria II (BD Biosciences, Franklin Lakes, NJ, United States) was employed to classify the macrophages from GC and non-tumor tissues.

Next, Ficoll-Paque Plus density gradient centrifugation was utilized to isolate the peripheral blood mononuclear cells (PBMCs) from both the healthy donors and GC patients. Later, anti-CD3 magnetic bead (Miltenyi Biotec, Miltenyi, Germany) was applied to purify CD3^+^ T cells from PBMCs.

### Immumohistochemical Staining

The paraffin-embedded sections (4 μm) were dewaxed, hydrated, and incubated with primary antibody rabbit anti-human PD-L1 (ab205921, 1:25, Abcam, Cambridge, MA, United States) overnight at 4°C, followed incubation with a secondary antibody goat anti-rabbit immunoglobulin G (IgG) (ab6785, 1:1000, Abcam) and horse radish peroxidase (HRP)-labeled streptomycin protein (0343-10000U, Yi Mo Biological Technology Co., Ltd., Beijing, China). Next the sections were visualized by diaminobenzidine (ST033, Whiga Biosmart Co., Ltd., Guangzhou, China), counterstained by hematoxylin (PT001, Shanghai Bogoo Biological Technology Co., Ltd., Shanghai, China) for 1 min, observed and photographed under a microscope.

### Reverse Transcription Quantitative Polymerase Chain Reaction

Total RNA was extracted in accordance with the instructions of the Trizol kit (Shanghai Haling Biotechnology Co., Ltd., Shanghai, China). Next, miRNA was reversely transcribed into complementary DNA (cDNA) as per the instructions of the TaqMan MicroRNA Assays Reverse Transcription prime kit, while mRNA was reversely transcribed into cDNA based on the method prescribed by the EasyScript First-Strand cDNA Synthesis SuperMix kit (AE301-02, Beijing TransGen Biotech Co., Ltd., Beijing, China). Fluorescent quantitative PCR was conducted based on the instructions of the SYBR^®^Premix Ex TaqTM II kit (TaKaRa, Tokyo, Japan), while reverse transcription quantitative polymerase chain reaction (RT-qPCR) was performed using 7500-type fluorescent quantitative PCR instrument (ABI Company, Oyster Bay, NY, United States). U6 was regarded as the loading control of miR-16-5p, while glyceraldehyde-3-phosphate dehydrogenase (GAPDH) was considered as the internal reference of PD-L1. All primers were synthesized by Beijing Genomics Institute, Co., Ltd., (Beijing, China) (Table 1). Quantitative analyses were conducted using the 2^–ΔΔCt^ method.

**TABLE 1:** Primer sequences for RT-qPCR.

Target	Primer sequence (5′-3′)
miR-16-5p	F: 5′-TCCACTCTAGCAGCACGTAAAT-3′
	R: 5′-TCACACTAAAGCAGCACAGTAAT-3′
PD-L1	F: 5′-CTCGCTTCGGCAGCACA-3′
	R: 5′-AACGCTTCACGAATTTGCGT-3′
U6	F: 5′-ATTGGAACGATACAGAGAAGATT-3′
	R: 5′-GGAACGCTTCACGAATTTG-3′
GAPDH	F: 5′-GTCAACGGATTTGGTCTGTATT-3′
	R: 5′-CGCUUCACGAAUUUGCGUGUCAU-3′

*F, forward; R, reverse.*

### Western Blot Analysis

Total protein was extracted from the tissues or cells. After isolation by means of polyacrylamide gel electrophoresis, the protein was transferred onto a polyvinylidene fluoride membrane. The membrane was subsequently incubated with primary antibodies (from Abcam) PD-L1 (ab205921, 1:25), inducible nitric oxide synthase (iNOS) (ab136918, 1:600), CD63 (ab216130, 1:1000), heat shock protein (HSP-70) (ab2787, 1:1000), and GAPDH (ab8245, 1:5000) overnight at 4°C. Next, the membrane was incubated with HRP-labeled goat anti-rabbit IgG (ab205718, 1:20000) at room temperature for 1.5 h. For iNOS, a surface marker of M1 macrophages, its membrane protein was extracted by the ProteoPrep^®^ Membrane Extraction Kit (Merck/Sigma-Aldrich). Finally, ImageJ 1.48u software was applied for protein quantitative analysis (Bio-Rad, Hercules, CA, United States). Membrane protein was normalized to Na/K ATPase (1:600, #3031, Cell Signaling Technology, Shanghai, China).

### Cell Treatment

The GC cell lines AGS and NCI-N87, purchased from Shanghai Institute of Cellular Research, Chinese Academy of Sciences (Shanghai, China), were incubated with DMEM (Gibco by Life technologies, Grand Island, NY, United States) containing 10% fetal bovine serum (FBS) and penicillin/streptomycin (Gibco by Life technologies, Grand Island, NY, United States) at 37°C with 5% CO_2_. After detachment using 0.25% trypsin, the cells were passaged (1:3) and inoculated into a 6-well plate (3 × 10^5^ cells/well). When cell confluence reached 70−80%, the cells in the logarithmic growth phase were collected for further treatment.

PBMCs were isolated using gradient centrifugation, and CD14^+^ mononuclear cells were purified by immunomagnetic beads. CD14^+^ mononuclear cells after purification were induced by granulocyte-macrophage colony stimulating factor for 5-day. Following medium change, M1 macrophages were induced by LPS (100 ng/ml) and IFN-γ (20 ng/ml). M1 macrophages were later inoculated into a 6-well plate (4 × 10^5^ cells/well) and transduced with miR-16-5p mimic and miR-16-5p inhibitor lentivirus (2 mL/well) at 37°C overnight. After 48 h, RT-qPCR was conducted in order to determine the relevant gene expression.

### Isolation and Identification of Exosomes Derived From Macrophages

M1-polarized macrophages were cultured with serum-free medium for 24 h, after which the supernatant was collected, followed by centrifugation at 2,000 × *g* and 4°C for 20 min. The supernatant was subjected to further centrifugation at 10,000 × *g* and 4°C for 1 h. The pellets were suspended by serum-free DMEM containing 25 mM 4-(2-hydroxyethyl)-1-piperazineethanesulfonic acid (pH = 7.4). The aforementioned centrifugation process was repeated again, after which the pellets were collected. Later, a transmission electron microscope (TEM) was utilized to identify exosomes (Cheng et al., 2017), after which the flow cytometer Guava easyCyteTM system together with CD63-phycoerythrin (PE) antibody was utilized to ascertain the level of the exosome surface marker CD63. The cells that were not subject to antibody addition were regarded as the blank control, while those added with PE-labeled anti-human IgG were considered to be the negative control (NC).

### Co-culture of M1 Macrophage-Secreted Exosomes With GC Cells

Exosome inhibitor GW4869 was employed to treat the macrophages with the objective of inhibiting the release of exosomes. The macrophages were settled on a 6-well plate (1 × 10^6^ cells/well). When cell confluence reached 80–90%, the cells were treated with 10% GW4869 (D1692-5MG, Sigma-Aldrich Chemical Company, St. Louis, MO, United States), while those treated with 0.005% dimethyl sulfoxide (DMSO) were regarded as the control. After 24 h, the cells and the supernatant were collected for subsequent use.

M1 macrophages were settled on the basolateral chamber of a 24-well transwell chamber (1 × 10^4^ cells/well), while the AGS cells (5 × 10^4^ cells/well) were covered on its apical chamber. The insert aperture between the apical and basolateral chambers was 0.4 μm. After 24 h of co-culture of AGS cells with macrophage-derived exosomes, AGS cells were isolated to evaluate the expression of miR-16-5p and PD-L1.

The macrophage-secreted exosomes were then labeled with PKH76 (green) dye liquor (MINI67-1KT, Sigma-Aldrich Chemical Company, St. Louis, MO, United States) and co-cultured with the supernatant of the AGS cells inoculated in the 24-well plate after their confluence had reached 50–60%. The co-cultured AGS/NCI-N87 cells were treated with exo-miR-16-5p mimic or exo-miR-16-5p inhibitor (the concentration of these exosomes was determined by BCA and appropriate concentration was selected according to experiments). RT-qPCR and western blot analysis were performed in order to determine the expression of miR-16-5p and PD-L1.

### Co-culture of GC Cells and T Cells *in vitro*

Carboxyfluorescein succinimidyl amino ester (CFSE)-labeled CD3^+^ T cells (10^5^ cells/96-well plate) were co-cultured with the AGS cells (10^3^ cells/96-well plate) treated with M1 macrophage-derived exosomes in the medium containing rhIL-2 (20 IU/mL), anti-CD3 (2 μg/mL), and anti-CD28 (1 μg/mL) antibodies. More specifically, the co-cultured cells were treated with M1 macrophage-derived exosomes, anti-PD-L1, exo-miR-16-5p mimic and exo-miR-16-5p inhibitor. After a period of 48 h had elapsed, the T cells were collected for flow cytometry, with the supernatant acquired to determine the expression of the cytokines using enzyme-linked immunoassay (ELISA).

### Flow Cytometry

The cells were made into single-cell suspension, and resuspended in dye buffer solution (BD Biosciences, Franklin Lakes, NJ, United States). AGS cells were incubated with APC-PD-L1 (#329708, Mouse, 1:50, Biolegend, San Diego, CA, United States). The T cells were incubated with PerCP-CD3 (#100326, Armenian Hamster, 1:100 Biolegend) and Pacific blue-IFNγ (#505817, Rat, 1:50, Biolegend) that was pre-fixed and pre-permeabilized. Cell apoptosis was assessed using a flow cytometer BD FACS CantoII (BD Immunocytometry Systems, Franklin Lakes, NJ, United States) and analyzed by Flow Jo software.

### Enzyme-Linked Immunoassay

Isolation of PBMCs from mouse peripheral blood was conducted using the Mouse Peripheral Blood Lymphocyte Separation Kit (Beijing Solarbio Science & Technology Co., Ltd., Beijing, China). Briefly, the mice were anesthetized with the eyeball obtained for blood collection. The blood was then quickly diluted with an equal volume of PBS, added with the separation solution, and subjected to gradient centrifugation to finally obtain the lymphocytes in peripheral blood. CD3^+^ T cells were then isolated by flow cytometry and cultured in the supernatant for 24 h. Cytokine expression was determined in accordance with the instructions of the ELISA kits including interleukin (IL)-2 (ab174444, Abcam, Cambridge, MA, United States), tumor necrosis factor (TNF)-α (ab181421, Abcam), and INF-γ (DIF50, R&D Systems, Minneapolis, MN, United States). A multifunctional microplate reader (Synergy^TM^ 2, Bio Tek Instruments, Winooski, VT, United States) was applied to measure the optical density (OD) value of each well at a wavelength of 450 nm. With the standard sample concentration as the abscissa, and the OD value as the ordinate, the regression equation of standard curve was calculated. The OD value was substituted into the equation to calculate target protein concentration.

### Dual Luciferase Reporter Gene Assay

Dual luciferase reporter gene vector 3′untranslated region (3′UTR) of PD-L1 (CD247) and mutant (MUT) plasmid mutated on its conjugated binding site with miR-16-5p were constructed, namely, PmirGLO-PD-L1-wild type (WT) and PmirGLO-PD-L1-MUT. The plasmids were co-transfected with miR-16-5p mimic or NC mimic into the HEK293T cells. After 24 h, the cells were lysed and centrifuged at 25,764 × *g* for 1 min. Dual-Luciferase^®^ Reporter Assay System (E1910, Promega Corporation, Madison, WI, United States) was employed to detect the luciferase activity. The ratio of firefly luciferase activity to renilla luciferase activity was regarded as the relative luciferase activity.

### Animal Studies

A total of 10 BALB/c mice (age: 4–6 weeks; weight: 16–18 g) were assigned into 2 groups (5 mice per group). Following the administration of pentobarbital sodium 50 mg/kg (57-33-0, Shanghai Beizhuo Biotechnology Co., Ltd., Shanghai, China), the mice were subcutaneously injected with AGS cells (1 × 10^6^ cells per mouse), and treated with phosphate buffer solution (PBS) or M1-exo suspension by tail injection. The tumor size and volume were evaluated every 2 days.

Additionally, a total of 30 female NOD/SCID mice (age: 5–7 weeks) were selected. AGS cells were incubated with exosomes and exosomes-containing miR-16-5p inhibitor for 48 h. Next, 100 μL of AGS cells (1 × 10^6^ cells per mouse) were subcutaneously injected into the armpit region after which the tumors were permitted to grow over a period of 10 days. Later, 5 × 10^6^ polyclone T cells [treated with 2 mg/mL anti-CD3 (ab135372, Abcam, Cambridge, MA, United States) and 1 mg/mL anti-CD28 (ab243228, Abcam, Cambridge, MA, United States)] were co-cultured with the AGS cells (1 × 10^6^ cells per mouse) and then with 5 μg/mL PD-L1 or 5 μg/mL control IgG for 24 h. The co-culture system of AGS cells and T cells were injected into the peritoneum of mice bearing 10-day subcutaneous tumors. The tumor size and volume were measured every 2 days. The mice were euthanized with 50 mg/kg pentobarbital sodium (57-33-0, Shanghai Beizhuo Biotechnology Co., Ltd., Shanghai, China), after which their respective tumors were removed, photographed and fixed for immumohistochemical staining, RT-qPCR and ELISA. The spleen was split into single cells for flow cytometry purposes as previously reported method (Wang et al., 2017). Additionally, tumor tissue supernatants followed by determination of expression of anti-tumor molecules perforin and granzyme B in the supernatants using ELISA kits according to the manufacturer’s instructions.

### Statistical Analysis

All data, expressed as mean ± standard deviation, were analyzed using a Statistic Package for the Social Science (SPSS) 21.0 statistical software (IBM Corp., Armonk, NY, United States). If departure from normal distribution and homogeneity of variance was not observed, comparisons between two groups were analyzed by means of a non-paired *t* test, while comparisons among multiple groups were analyzed by one-way analysis of variance (ANOVA) with Tukey’s *post hoc* test, while date comparisons at various time points were analyzed by repeated measures ANOVA with Bonferroni’s *post hoc* test. Kaplan–Meier method was applied in order to calculate the OS of the GC patients, and Log-rank test was applied for single factor analysis. Significant difference was reflected by *p* < 0.05.

## Results

### Enrichment of M1 Macrophages Was Associated With GC Progression

**FIGURE 1:**
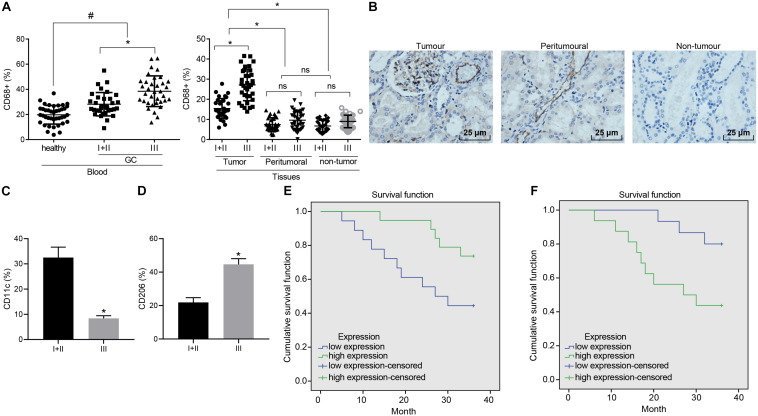
Enrichment of M1 macrophages is associated with GC progression. **(A)** Expression of CD68^+^ macrophages in peripheral blood of GC patients and healthy donors, cancerous, tumor-adjacent, and non-cancerous stomach tissues detected by flow cytometry (healthy donors: *n* = 40; GC patients: *n* = 68). **(B)** Immunohistochemical staining of CD68^+^ macrophages in cancerous, tumor-adjacent, and non-cancerous stomach tissues (400×). **(C)** The ratio of CD68^+^ M1 macrophages (CD11c) in patients at different stages assessed by flow cytometry. **(D)** The ratio of CD68^+^ M2 macrophages (CD206) in patients at different stages assessed by flow cytometry. **(E)** Kaplan–Meier survival analysis of the correlation between M1 macrophages (CD11c) and OS of GC patients. **(F)** Kaplan–Meier survival analysis of the correlation between M2 macrophages (CD206) and OS of GC patients. Measurement data representative of three independently performed experiments expressed as mean ± standard deviation. Comparisons between two groups are analyzed by non-paired *t* test, with comparisons among multiple groups are analyzed by one-way ANOVA followed by Tukey’s test. Kaplan–Meier method is applied to test the OS of GC patients. *p < 0.05 compared with stage I and stage II gastric cancer patients; ^#^*p* < 0.05 compared with healthy volunteers.

To elucidate the role of macrophages in GC, enrichment of the macrophages at various stages was analyzed in different samples. Initially, flow cytometry was performed to evaluate its enrichment in peripheral blood. The ratio of CD68^+^ macrophages in peripheral blood of the GC patients was observed to be higher than that of the healthy donors. Next, enrichment in the GC tissues was also revealed that the ratio of CD68^+^ macrophages in the GC tissues was greater than that in the tumor-adjacent and non-cancerous stomach tissues. As depicted in Figure 1A, the ratio of macrophages in each sample was elevated with the development of GC. Similarly, immumohistochemical staining further illustrated a large number of macrophages were accumulated in GC tissues (Figure 1B), highlighting the potential involvement of macrophages in the GC microenvironment.

Flow cytometry was further conducted to assess the ratio of CD68^+^ macrophages in M1 macrophages (CD11c) and M2 macrophages (CD206) in patients at different stages. Our results revealed that as GC deteriorated, the ratio of M1 macrophages decreased from 32 to 8% (Figure 1C), while the ratio to M2 macrophages was elevated from 22 to 44% (Figure 1D). The Kaplan–Meier method demonstrated that poor expression of CD11c or high expression of CD206 was linked to poor OS (Figures 1E,F).

### M1 Macrophage-Derived Exosomes Inhibited GC Progression

**FIGURE 2:**
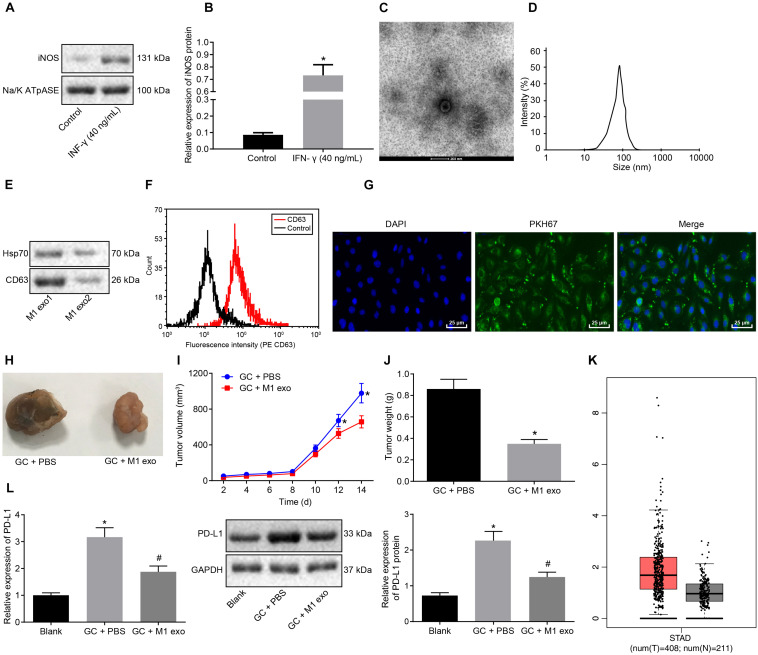
Exosomes derived from M1 macrophages suppress GC progression. **(A)** Western blot analysis of the cell surface marker iNOS protein expression in LPS (100 ng/ml) + IFN-γ (20 ng/ml)-induced macrophages (M1 macrophages) normalized to Na/K ATPase. **(B)** M1 macrophage membrane proteins quantified by image J and normalized with Na/K ATPase as well as statistics of iNOS gray value. **(C)** TEM observation of M1 macrophage-secreted exosomes (5,000×). **(D)** Dynamic light scattering detection of the diameter of exosomes. **(E)** Western blot analysis of the exosome surface markers (CD63 and HSP70) protein expression in M1 macrophages. **(F)** Flow cytometry of the exosome surface marker CD63 level. **(G)** Uptake of PKH76-labeled exosomes by GC cells was analyzed under a confocal fluorescence microscope (400×). **(H)** Tumor morphology in model mice with GC (*n* = 5). **(I)** Tumor volume in model mice with GC (*n* = 5). **(J)** Tumor weight in model mice with GC (*n* = 5). **(K)** Box plots of PDL1 expression in GC samples from TCGA analyzed by GEPIA (http://gepia.cancer-pku.cn/), wherein the left is GC tissues and the right is normal tissues, and the vertical axis is log2 [tags per million (TPM) + 1] (all PDL1 levels were normalized by TPM). **(L)** Western blot analysis of PD-L1 protein expression in model mice with GC normalized to GAPDH after protein quantitation by Image J (*n* = 5). *p < 0.05 compared with macrophages without the induction of IFN-γ or M1 exosome or mouse GC models without any treatment; #*p* < 0.05 compared with mouse GC models treated with PBS. Measurement data was representative of three independently conducted experiments and expressed as the mean ± standard deviation. Comparisons between two groups are analyzed by non-paired *t* test, and comparisons among multiple groups are analyzed by one-way ANOVA. *Post hoc* test is conducted using Tukey’s test.

To further investigate the effects associated with M1 macrophages on GC progression, PBMCs-induced macrophages were induced into M1 macrophages by LPS (100 ng/ml) and IFN-γ (40 ng/mL) *in vitro*. Membrane protein was extracted by the Membrane Protein Extraction Kit. Western blot analysis was conducted to determine the expression of cell surface marker iNOS with Na/KATPase used as an internal reference (Figures 2A,B). Our results revealed that the expression of iNOS was elevated after LPS (100 ng/ml) and IFN-γ (20 ng/ml) induction. Next, exosomes were isolated from M1 macrophages. TEM observed that the M1 macrophage-derived exosomes had uniformly circular or oval-shaped membranous vesicles (Figure 2C). Dynamic light scattering revealed that their size ranged from 30 to 120 nm (Figure 2D). Western blot analysis displayed that the expression of the exosome surface markers (CD63 and HSP70) was higher in the aforementioned exosomes (Figure 2E). Besides, the level of CD63 was increased as reflected by flow cytometry, suggesting that exosomes had been successfully extracted (Figure 2F).

Next, to further determine whether the GC cells could absorb M1 macrophage-secreted exosomes, PKH76 (Green)-labeled exosomes were co-cultured with GC cells for 48 h. The uptake of PKH76-labeled exosomes by GC cells was analyzed under a confocal fluorescence microscope (Figure 2G). After 48 h of co-culture, the GC cells were noted to exhibit a green fluorescence, while a distinct uptake of PKH76-labeled exosomes by the GC cells was identified, indicating that exosomes could transfer from donor M1 macrophages to receptor GC cells. Besides, the above assays were repeated in NCI-N87 cells, from which the same results were obtained (Supplementary Figures 1, 2).

After the mice were treated with 10 μg M1 macrophage-secreted exosomes via tail vein injection, the tumor growth, volume and weight of the mice decreased *in vivo* (Figures 2H–J). The GEPIA database^1^ found that PDL1 was upregulated in the GC samples from TCGA (Figure 2K). Next, the effect of M1 macrophages on PD-L1 in GC microenvironment was explored. As reflected by RT-qPCR and western blot analysis, the expression of PD-L1 was increased in the tumors of the GC mice and reduced in those treated with M1 macrophage-secreted exosomes (Figure 2L). Therefore, we concluded that the M1 macrophage-secreted exosomes could inhibit GC progression and down-regulate PD-L1 expression.

### M1 Macrophage-Secreted Exosomes Enhanced Immune Response of T Cells in GC

**FIGURE 3:**
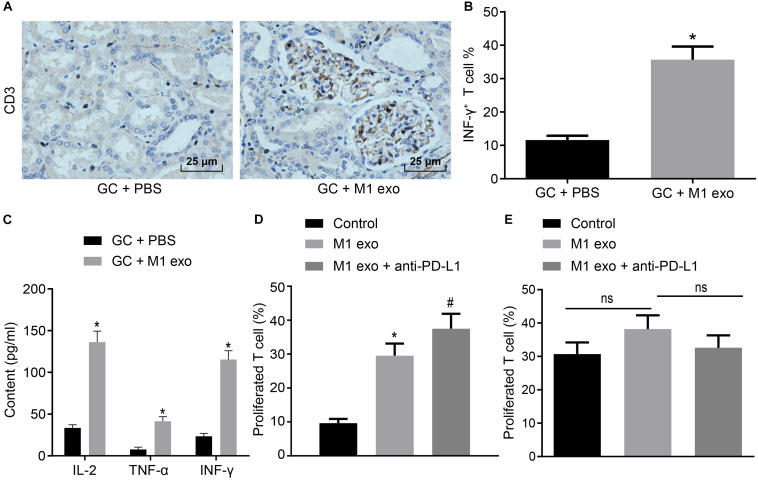
Exosomes derived from M1 macrophages promote the immune response of T cells. Mice are treated with PBS or M1 macrophage-derived exosomes. **(A)** Immunohistochemistry of CD3^+^ T infiltration in the tumors of mice (400×). **(B)** Flow cytometry of INF-γ^+^ T cells in CD3^+^ T cells (*n* = 5). **(C)** ELISA of IL-2, TNF-α and INF-γ expression in the supernatant of T cells (*n* = 5). **(D)** Flow cytometry of PD-1^+^ T cell proliferation in GC cells treated with M1 macrophage-derived exosomes and anti-PD-L1 after co-cultured with PD-1^+^ or PD-1^–^ T cells. **(E)** Flow cytometry of PD-1^–^ T cell proliferation in GC cells treated with M1 macrophage-derived exosomes and anti-PD-L1 after co-cultured with PD-1^–^ T cells or PD-1^–^ T cells. *p < 0.05 compared to mice treated with PBS as well as in comparison to the GC cells without any treatment; #*p* < 0.05 compared with GC cells treated with M1 macrophage-secreted exosomes. Measurement data representative of three independently conducted experiments expressed as mean ± standard deviation. Comparisons between two groups analyzed by non-paired *t* test as well as comparisons among multiple groups are analyzed by one-way ANOVA. *Post hoc* test was conducted using Tukey’s test.

M1 macrophages can inhibit disease progression by enhancing the T cell immune response (Cheng et al., 2017). Meanwhile, elevated PD-L1 expression in GC can stimulate the immune tolerance of T cells (Lazar et al., 2018). Thus, it could be speculated that M1 macrophage-secreted exosomes could repress the tumor growth in GC by inducing the T cell immune response. To verify this hypothesis, immunohistochemistry was conducted to detect CD3^+^ T cell inflammation in the tumors of mice, the result of which revealed that CD3^+^ T cell inflammation was enhanced in those treated with M1 macrophage-secreted exosomes (Figure 3A). Next, CD3^+^ T cells were isolated from the tumors. Flow cytometry was conducted to assess the INF-γ^+^ T cells in CD3^+^ T cells (Figure 3B). The results obtained provided evidence demonstrating that T cell activation enhanced in mice treated with M1 macrophage-secreted exosomes, suggesting that M1 macrophage-secreted exosomes could activate T cells. In the following, ELISA was performed to determine the expression of IL-2, TNF-α and INF-γ in the supernatant of T cells (Figure 3C). The results demonstrated that the expression of these factors was elevated after the mice treated with M1 macrophage-secreted exosomes, revealing that M1 macrophage-secreted exosomes could enhance the immune response of T cells.

Subsequently, to study the interactive impact of PD-L1 and PD-1 on T cell activation, GC cells treated with M1 macrophage-derived exosomes were added with anti-PD-L1 after co-cultured with PD-1^+^ and PD-1^–^ T cells. Based on the results from flow cytometry (Figures 3D,E), the treatment of the M1 macrophage-derived exosomes were found to have potentiated PD-1^+^ T cell proliferation. The treatment of M1 macrophage-derived exosomes and anti-PD-L1 promoted PD-1^+^ T cell proliferation, however, it did not alter PD-1^–^ T cell proliferation. Based on the aforementioned results, we concluded that M1 macrophage-derived exosomes could activate the immune response of T cells by inhibiting PD-L1 expression.

### M1 Macrophage-Secreted Exosomes Carrying miR-16-5p Reduced PD-L1 Expression in GC

**FIGURE 4:**
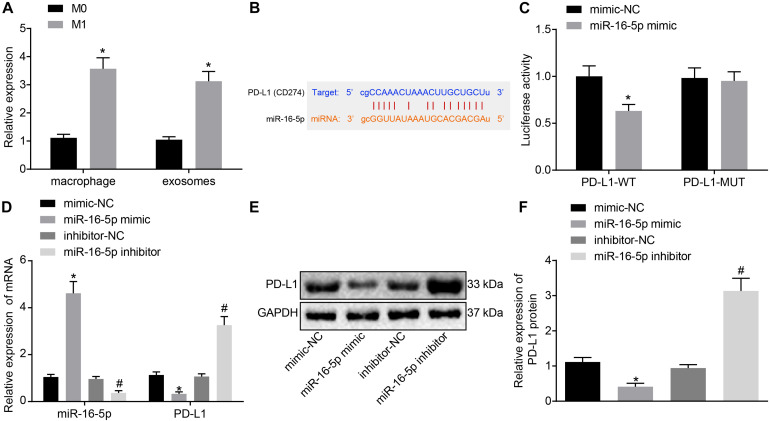
Exosomes derived from M1 macrophages harboring miR-16-5p repress PD-L1 expression. **(A)** RT-qPCR of miR-16-5p expression in unpolarized M0-, M1-polarized macrophages and their secreted exosomes. **(B)** Starbase (http://starbase.sysu.edu.cn) prediction of binding site between miR-16-5p and PD-L1. **(C)** Dual luciferase reporter gene assay verification of the target relationship between miR-16-5p and PD-L1. **(D)** RT-qPCR of PD-L1 expression in macrophages treated with miR-16-5p mimic and miR-16-5p inhibitor. **(E)** Western blot analysis of PD-L1 expression in macrophages treated with miR-16-5p mimic or miR-16-5p inhibitor. **(F)** Statistic analysis of PD-L1 expression in macrophages treated with miR-16-5p mimic or miR-16-5p inhibitor; mimic-NC: negative control for miRNA mimic; inhibitor-NC: negative control for miRNA inhibitor. *p < 0.05 versus unpolarized M0 macrophages and its secreted exosomes or cells treated with mimic-NC; #*p* < 0.05 compared to cells treated with inhibitor-NC. Measurement data representative of three independently conducted experiments expressed as the mean ± standard deviation. Comparisons between two groups are analyzed by non-paired *t* test.

Existing literature has suggested that miR-16-5p can inhibit PD-L1 expression and induce the polarization of macrophages to its M1 phenotype (Jia et al., 2016). Hence, we speculated that high levels of miR-16-5p expression in M1 macrophage-secreted exosomes may inhibit PD-L1 expression in GC. First, RT-qPCR confirmed that compared with unpolarized M0-macrophages, miR-16-5p expression increased in M1 macrophage and its secreted exosomes (Figure 4A). Next, to evaluate the regulatory mechanism associated with miR-16-5p and PDL1 in exosomes, Starbase^2^ provided data predicting the existence of a binding site between miR-16-5p and PDL1 (Figure 4B), which was subsequently verified by dual luciferase reporter gene assay that in the presence of miR-16-5p mimic, the luciferase activity of PD-L1-WT decreased (*p* < 0.05), while no significant difference was detected in relation to PD-L1-MUT (*p* > 0.05) (Figure 4C), suggesting that miR-16-5p could specifically bind to PD-L1. Moreover, as reflected by RT-qPCR and western blot analysis, PD-L1 expression was weakened in M1 macrophages by miR-16-5p mimic and strengthened by miR-16-5p inhibitor (Figures 4D–F). Therefore, PD-L1 expression was negatively regulated by miR-16-5p.

### M1 Macrophage-Derived Exosomes Carrying miR-16-5p Promoted the Activation of T Cells in the Co-culture System With GC Cells

**FIGURE 5:**
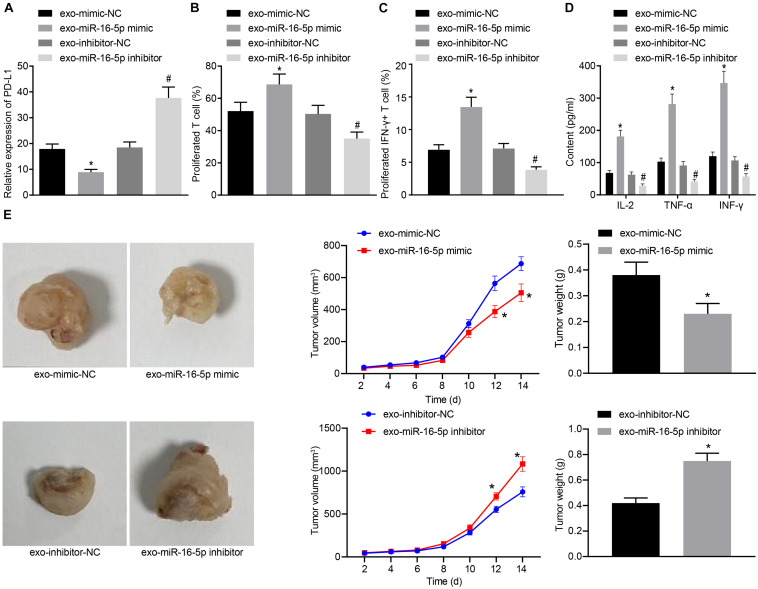
Exosomes secreted from M1 macrophages harboring miR-16-5p promote the activation of T cells in the co-culture system with GC cells. **(A)** Flow cytometry of PD-L1 expression in GC cells treated with exo-miR-16-5p mimic and exo-miR-16-5p inhibitor. **(B)** Flow cytometry of CD3^+^ T cell proliferation in GC cells co-cultured with T cells for 24 h after treated with exo-miR-16-5p mimic or exo-miR-16-5p inhibitor. **(C)** Flow cytometry of the number of INF-γ^+^ T cells in GC cells co-cultured with T cells for 24 h after treated with exo-miR-16-5p inhibitor or exo-miR-16-5p inhibitor. **(D)** ELISA was performed to determine levels of IL-2, TNF-α and INF-γ in the supernatant of T cells after treated with exosomes. **(E)** Representative images of tumors in mice after tail injection of GC cells stably transfected with exo-miR-16-5p mimic or exo-miR-16-5p inhibitor as well as quantification of tumor volume and weight (*n* = 5). *p < 0.05 cells treated with exo-mimic-NC; #*p* < 0.05 compared with cells treated with exo-inhibitor-NC. Measurement data representative of three independently conducted experiments expressed as the mean ± standard deviation. Comparisons between two groups are analyzed by non-paired *t* test.

In the following, GC cells treated with M1 macrophage-derived exosomes were co-cultured with T cells. After GC cells were treated with exo-miR-16-5p mimic and exo-miR-16-5p inhibitor, flow cytometry was conducted to determine PD-L1 expression (Figure 5A). Key observations were made indicating that PD-L1 expression was decreased following treatment with exo-miR-16-5p mimic and increased by the treatment of exo-miR-16-5p inhibitor. After the co-culture of GC cells and CD3^+^ T cells, flow cytometry was employed to assess CD3^+^ T cell proliferation and the number of INF-γ^+^ T cells. As depicted in Figures 5B,C, in the cells treated with exo-miR-16-5p mimic, enhanced CD3^+^ T cell proliferation as well as an increased number of INF-γ^+^ T cells were detected, while contrasting results were identified in cells treated with exo-miR-16-5p inhibitor. ELISA found that the introduction of exo-miR-16-5p mimic facilitated the expression of IL-2, TNF-α and INF-γ, while the introduction of exo-miR-16-5p inhibitor blocked their expression (Figure 5D). Next, mouse models of GC were treated with exo-mimic-NC, exo-miR-16-5p mimic, exo-inhibitor-NC, and exo-miR-16-5p inhibitor by tail injection, respectively, to detect the effect of miR-16-5p in the exosomes on GC progression. The results displayed that up-regulation of miR-16-5p reduced the tumor growth rate, volume, and weight, while silencing miR-16-5p led to the opposite trends (Figure 5E). Thus, M1 macrophage-derived exosomes carrying miR-16-5p were confirmed to inhibit PD-L1 expression and trigger T cell activation, thereby inhibiting GC development.

### M1 Macrophage-Secreted Exosomes Inhibited Anti-tumor Immune Response and Tumor Formation *in vivo* by Repressing PD-L1 Expression

**FIGURE 6:**
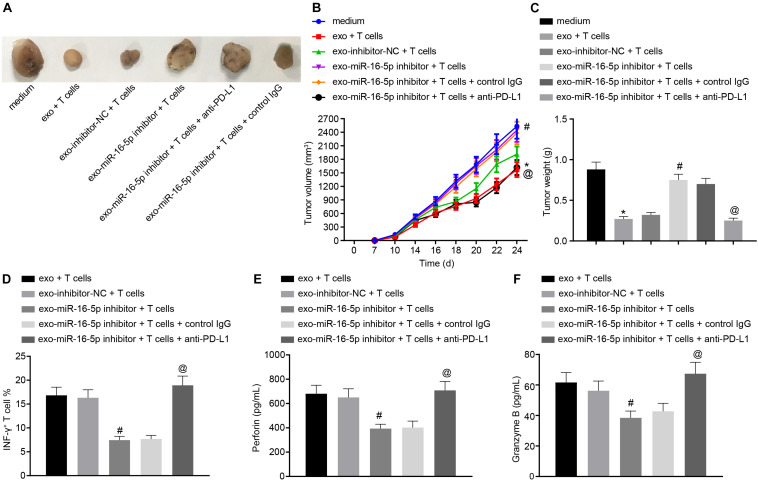
M1 macrophage-secreted exosomes containing miR-16-5p impair anti-tumor immune response and tumor formation *in vivo* by down-regulating PD-L1. The mice were injected with GC cells (5 × 10^6^ cells per mouse) and T cells (1 × 10^6^ cells per mouse) for 24-h incubation. **(A)** Tumor morphology in mice (*n* = 5). **(B)** Tumor volume in mice (*n* = 5). **(C)** Tumor weight in mice (*n* = 5). **(D)** Flow cytometry of activated INF-γ^+^ T cells in the spleen cells. **(E)** EILSA of the expression of perforin in lysis buffer of the spleen cells (*n* = 5). **(F)** EILSA of the expression of granzyme B in lysis buffer of the spleen cells (*n* = 5). *p < 0.05 compared with the treatment of medium; #*p* < 0.05 compared with the treatment of exo-inhibitor-NC + T cells. @*p* < 0.05 compared with the treatment of exo-miR-16-5p inhibitor + T cells + Control IgG or exo-miR-16-5p inhibitor + T cells. Measurement data is representative of three independently performed experiments and expressed as the mean ± standard deviation. Comparisons among multiple groups are analyzed by one-way ANOVA with Tukey’s *post hoc* test. Data comparisons at various time points are analyzed by repeated measures ANOVA with Bonferroni’s *post hoc* test. *n* = 5.

To further investigate the effect of M1 macrophage-secreted exosomes carrying miR-16-5p on GC progression *in vivo*, the mice were initially injected with GC cells treated with M1 macrophage-secreted exosomes and subsequently with T cells (2 mg/mL anti-CD3 and 1 mg/mL anti-CD28) co-cultured with GC cells. After assessment, we found that relative to the treatment of medium, the tumor growth, volume and weight were impeded by the treatment of M1 macrophage-secreted exosomes and T cells. When compared with matched controls, the tumor growth, volume and weight were increased by the treatment of exo-miR-16-5p inhibitor and T cells and decreased by the treatment of exo-miR-16-5p inhibitor, T cells and anti-PD-L1 (Figures 6A–C), suggesting that M1 macrophage-secreted exosomes carrying miR-16-5p inhibited tumor formation by repressing PD-L1 expression *in vivo*. The above assays were also conducted in NCI-N87 cells and the same results were obtained.

Next, to detect T cell activation in the tumors of the mice, flow cytometry was conducted to evaluate cell suspension of the spleen with the results indicated that the INF-γ^+^ T cells were reduced following treatment with the exo-miR-16-5p inhibitor and T cells while an increase was detected following the treatment of exo-miR-16-5p inhibitor, T cells and anti-PD-L1 (Figure 6D). Meanwhile, EILSA was utilized to examine the expression of anti-tumor molecules perforin and granzyme B in lysis buffer of the spleen cells. The results demonstrated that the treatment of exo-miR-16-5p inhibitor and T cells decreased the expression of perforin and granzyme B, while the treatment of exo-miR-16-5p inhibitor, T cells and anti-PD-L1 led to a contrasting trend of results (Figures 6E,F). Therefore, M1 macrophage-secreted exosomes carrying miR-16-5p inhibited PD-L1 expression to activate the immune response of T cells, thus suppressing tumor formation *in vivo*.

## Discussion

The reported survival rates in the advanced stages of GC remain poor. In a previous pembrolizumab clinical trial targeting PDL1, the tumors in more than 50% of patients with advanced stage GC shrunk from baseline, highlighting the promising outcomes associated with GC immunotherapy (Matsueda and Graham, 2014). During the current study, we investigated another form of immunotherapy mediated by exosome-derived miRNA targeting a key finding of our study revealed that M1 polarized macrophages were able to generate exosomes carrying miR-16-5p which subsequently targeted and downregulated the expression of PDL1 of GC cells. The downregulation of PDL1 was found to be capable of diminishing GC immune evasion from T cells monitor and activate the immune effects of T cells.

Initially, we identified diminished M1 macrophage numbers in relation to GC tumor growth with exosomes secreted by M1 macrophages found to suppress the proliferation of GC cells. There are two types of macrophages including M1 and M2. M1 macrophages represent the classically activated macrophages, which are key effector cells capable of eliminating infectious pathogens and cancer cells. Alternatively, M2 macrophages when activated are responsible for wound-healing and immune regulation (Italiani and Boraschi, 2014). However, existing literature has suggested that ornithine produced by M2 macrophages can promote cellular proliferation and repair collagen synthesis as well as remodel regional tissue, suggesting that M2 macrophages play a stimulatory role in tumor progression (Italiani and Boraschi, 2014). This was consistent with the findings of our study whereby M1 macrophages were reduced while M2 macrophages exhibited elevated numbers in line with GC progression. Similarly, one study shows that M2 macrophages are enriched in gastric and breast cancer tissues and promote metastasis of cancer cells mediated by CHI3L1 protein (Chen X. et al., 2017).

Secondly, the exosomes secreted by M1 macrophages were demonstrated to inhibit GC cells proliferation. Exosomes represent bio membrane vesicles capable of loading macro biomolecules that participate in a functional manner in cellular communications. In the midst of inflammation and immune responses, exosomes have been reported to present antigens and act as a shuttle for RNA and immune factors that regulate the surrounding immune cells (Schorey and Bhatnagar, 2008). A previous study reported that M1-derived nanovesicles could repolarize M2 tumor-associated macrophages to M1 type which ultimately enhances immune checkpoint inhibitor therapy against tumor growth (Choo et al., 2018). Likewise, in melanoma therapy, M1 as opposed to that of M2-derived exosomes represent an excellent vaccine adjuvant capable of potentiating vaccine effects by increasing proinflammatory responses (Cheng et al., 2017). The results of the present study illustrated that miR-16-5p plays a role as a functional molecule regulating PDL1 in tumor cells. The regulation between macrophages and tumor cells through exosomal miRNAs is a common way. For example, miR-501-3p loaded by M2-derived exosomes has been shown to downregulate TGFBR3, thus activating the TGF-β signaling pathway and promoting the progression of pancreatic ductal adenocarcinoma (Yin et al., 2019). Furthermore, cancer-derived exosomes have been demonstrated to influence macrophages in a reverse manner. Hypoxic epithelial ovarian cancer cells have also been shown to generate exosomes carrying miR-940 to promote M1 to M2 polarization (Chen Y. et al., 2017). The aforementioned data highlights the effectiveness of exosomal miRNAs as regulators between immune and cancer cells.

Finally, the miR-16-5p carried by exosomes was found to be sufficient to activate T cells responses to GC cells. Although the target relation between miR-16 and PDL1 has been reported in prostate cancer (Tao et al., 2018), we first identified the miR-16 in tumor microenvironment was loaded on M1-derived exosomes. The PD1/PDL1 checkpoint is generally utilized by cancer cells as a means of escaping T cell surveillance. In clinical therapy, dozens of antibodies have been designed to target PD1 or PDL1, and have shown an encouraging capacity to block the interaction between the two molecules. However, the side effects associated with artificial antibodies are still significant enough to limit their clinical application in humans (Sood et al., 2018). In contrast, exosomes structure owning double lipid membrane can reduce immunogenicity, which can also easily transfer molecules to target cells, highlighting the promise associated with developing exosome-based drugs that function as inhibitors of checkpoints to activate T cell immune responses and achieve successful immunotherapy.

## Conclusion

Taken together, the key findings of the present study indicate that M1 macrophages are negatively associated with GC progression, highlighting the anti-tumor role of M1 macrophages. M1 macrophages are able to generate exosomal miR-16-5p that specifically targeted and downregulated PDL1 on GC cells. Blockade of PD1/PDL1 checkpoints could lead to T cells activation and inhibit GC proliferation. However, the downstream signaling pathway after miR-16-5p blocking PD1/PDL1 checkpoints still requires further exploration in order to fully elucidate the mechanism.

## Data Availability Statement

The original contributions presented in the study are included in the article/Supplementary Material, further inquiries can be directed to the corresponding author.

## Ethics Statement

The studies involving human participants were reviewed and approved by the Ethics Committee of The First Affiliated Hospital of Harbin Medical University. The patients/participants provided their written informed consent to participate in this study. The animal study was reviewed and approved by the Institutional Animal Care and Use Committee of The First Affiliated Hospital of Harbin Medical University.

## Author Contributions

ZL, BS, DW, JS, MZ, and BF designed the study. ZL, BS, and DW collated the data, carried out data analyses, and produced the initial draft of the manuscript. JS, MZ, and BF contributed to drafting the manuscript. GL, YG, and CS contributed substantially to its revision. All authors have read and approved the final submitted manuscript.

## Conflict of Interest

The authors declare that the research was conducted in the absence of any commercial or financial relationships that could be construed as a potential conflict of interest.

## Footnotes

1 http://gepia.cancer-pku.cn/

2 http://starbase.sysu.edu.cn

## Supplementary Material

Supplementary Figure 1 **(A)** The absorption of exosomes into NCI-N87 cells observed by an inverted microscope (200×). **(B)** Representative images of tumors in mice after tail injection of NCI-N87 cells co-cultured with GC or M1 exo as well as tumor volume and weight (*n* = 5). **(C)** The expression of PD-L1 in the tumor of mice in different groups detected by Western blot analysis (*n* = 5). ^∗^*p* < 0.05 versus Control or M1 exo or GC + PBS or blank groups. **(D)** The expression of cytokines IL-2, TNF-α, and INF-γ in mice detected by ELISA (*n* = 5). **(E)** Representative images of tumors in mice after treatment of exo, T cells, exo-miR-16-5p inhibitor, or anti-PD-L1 as well as quantification of tumor volume and weight (*n* = 5). The data in the figure were measurement data, expressed as mean ± standard deviation. Unpaired *t*-test was used for comparison between two groups, and one-way ANOVA was used for comparison of data between multiple groups followed by Tukey’s *post hoc* test. Data between groups at different time points were compared using repeated measures ANOVA followed by Bonferroni’s *post hoc* test.

Supplementary Figure 2 **(A)** The expression of PD-L1 on the surface of NCI-N87 cells after co-culture with different groups of exosomes detected by flow cytometry. **(B)** The proliferation of T cells after co-culture of NCI-N87 cells treated with different groups of exosomes with T cells for 24 h detected by flow cytometry. **(C)** The number of activated INF-γ^+^ T cells after co-culture of NCI-N87 cells treated with different groups of exosomes with T cells detected by flow cytometry for 24 h. **(D)** The expression of cytokines in the supernatant of NCI-N87 cells co-cultured with T cells in different groups detected by ELISA. ^∗^*p* < 0.05 versus exo-mimic-NC group; #*p* < 0.05 versus exo-inhibitor-NC.
