# Supplementary figures and images for: Exosomal miRNA-16-5p Derived From M1 Macrophages Enhances T Cell-Dependent Immune Response by Regulating PD-L1 in Gastric Cancer
Source: Front Cell Dev Biol. 2020 Nov 30;8:572689. doi: 10.3389/fcell.2020.572689 (PMC7734296; doi:10.3389/fcell.2020.572689)

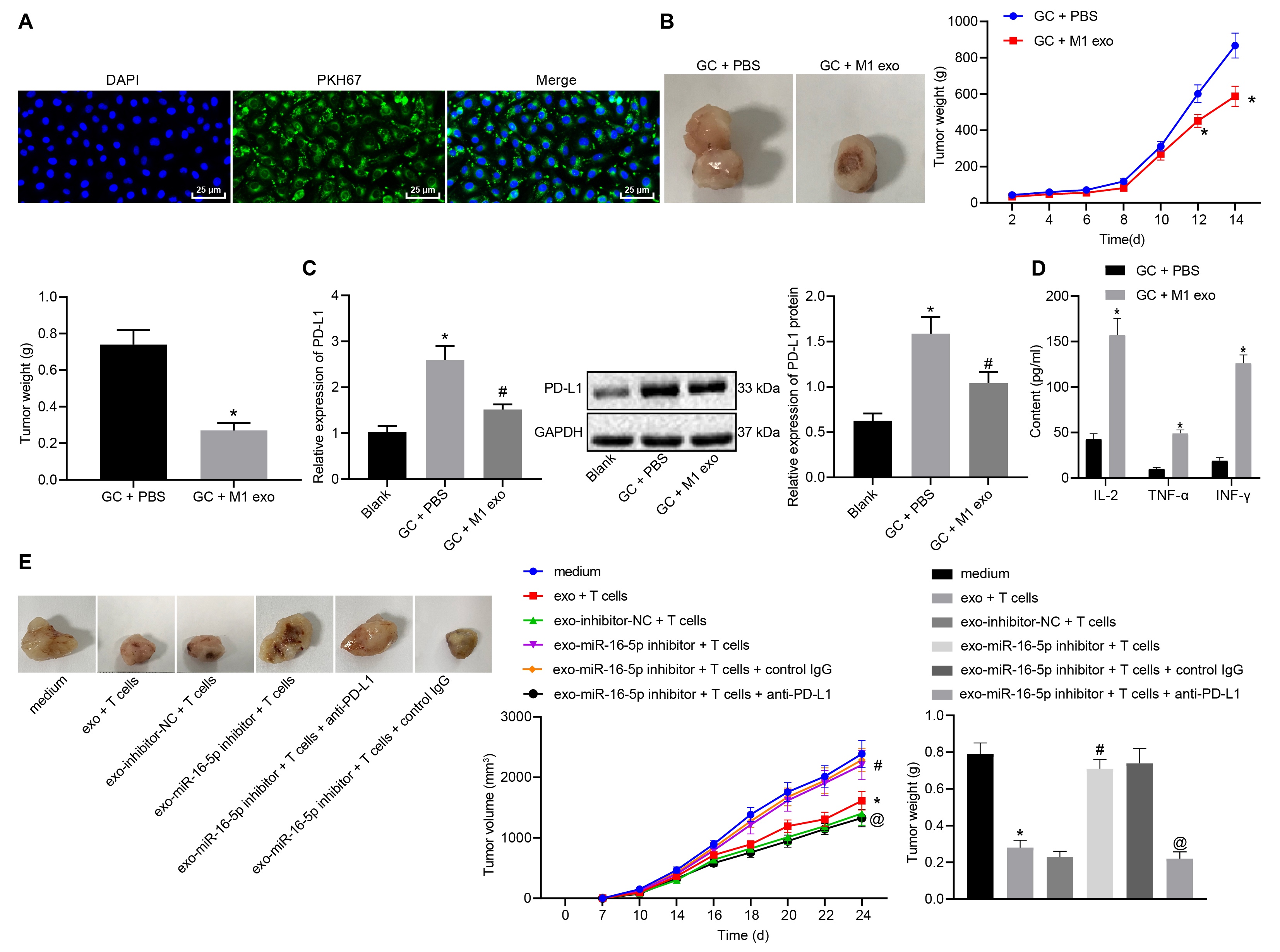

Supplement: Supplementary Figure 1 — (A) The absorption of exosomes into NCI-N87 cells observed by an inverted microscope (200×). (B) Representative images of tumors in mice after tail injection of NCI-N87 cells co-cultured with GC or M1 exo as well as tumor volume and weight (n = 5). (C) The expression of PD-L1 in the tumor of mice in different groups detected by Western blot analysis (n = 5). ∗p < 0.05 versus Control or M1 exo or GC + PBS or blank groups. (D) The expression of cytokines IL-2, TNF-α, and INF-γ in mice detected by ELISA (n = 5). (E) Representative images of tumors in mice after treatment of exo, T cells, exo-miR-16-5p inhibitor, or anti-PD-L1 as well as quantification of tumor volume and weight (n = 5). The data in the figure were measurement data, expressed as mean ± standard deviation. Unpaired t-test was used for comparison between two groups, and one-way ANOVA was used for comparison of data between multiple groups followed by Tukey’s post hoc test. Data between groups at different time points were compared using repeated measures ANOVA followed by Bonferroni’s post hoc test. [file Image_1.JPEG]

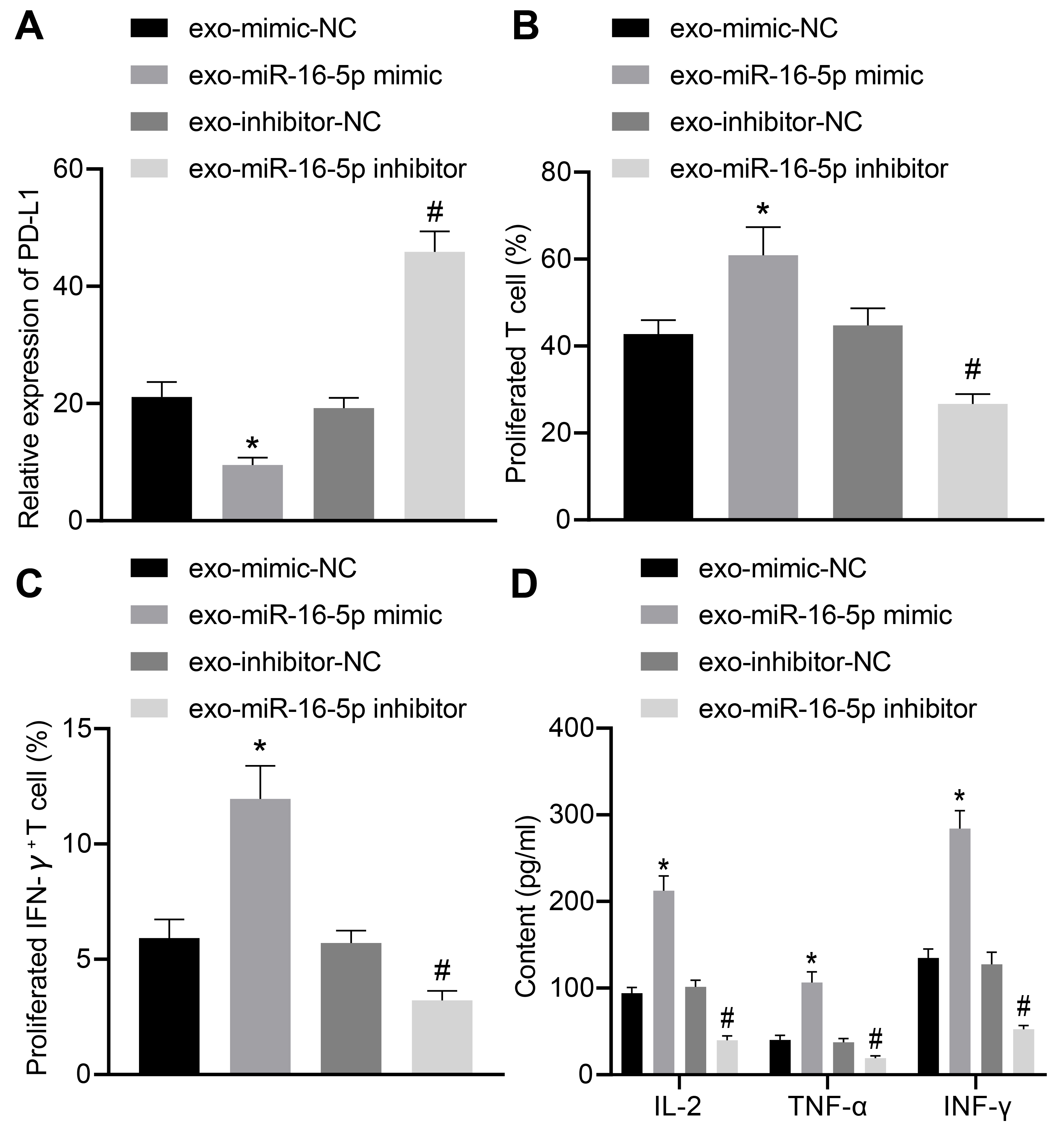

Supplement: Supplementary Figure 2 — (A) The expression of PD-L1 on the surface of NCI-N87 cells after co-culture with different groups of exosomes detected by flow cytometry. (B) The proliferation of T cells after co-culture of NCI-N87 cells treated with different groups of exosomes with T cells for 24 h detected by flow cytometry. (C) The number of activated INF-γ+ T cells after co-culture of NCI-N87 cells treated with different groups of exosomes with T cells detected by flow cytometry for 24 h. (D) The expression of cytokines in the supernatant of NCI-N87 cells co-cultured with T cells in different groups detected by ELISA. ∗p < 0.05 versus exo-mimic-NC group; #p < 0.05 versus exo-inhibitor-NC. [file Image_2.JPEG]
